# Three-phase junction for modulating electron–hole migration in anatase–rutile photocatalysts†
†Electronic supplementary information (ESI) available: Optimized structures (*XYZ* coordinates) for pure phase and biphase crystals; other structure models for phase junction. See DOI: 10.1039/c5sc00621j


**DOI:** 10.1039/c5sc00621j

**Published:** 2015-04-07

**Authors:** Wei-Na Zhao, Sheng-Cai Zhu, Ye-Fei Li, Zhi-Pan Liu

**Affiliations:** a Collaborative Innovation Center of Chemistry for Energy Material , Shanghai Key Laboratory of Molecular Catalysis and Innovative Materials , Key Laboratory of Computational Physical Science (Ministry of Education) , Department of Chemistry , Fudan University , Shanghai 200433 , China . Email: zpliu@fudan.edu.cn

## Abstract

The heterophase solid–solid junction as an important type of structure unit has wide applications for its special mechanics and electronic properties. Here we present a first three-phase atomic model for the anatase–rutile TiO_2_ heterophase junction and determine its optical and electronic properties, which leads to resolution of the long-standing puzzles on the enhanced photocatalytic activity of anatase–rutile photocatalysts. By using a set of novel theoretical methods, including crystal phase transition pathway sampling, interfacial strain analysis and first principles thermodynamics evaluation of holes and electrons, we identify an unusual structurally ordered three-phase junction, a layer-by-layer “T-shaped” anatase/TiO_2_-II/rutile junction, for linking anatase with rutile. The intermediate TiO_2_-II phase, although predicted to be only a few atomic layers thick in contact with anatase, is critical to alleviate the interfacial strain and to modulate photoactivity. We demonstrate that the three-phase junction acts as a single-way valve allowing the photogenerated hole transfer from anatase to rutile but frustrating the photoelectron flow in the opposite direction, which otherwise cannot be achieved by an anatase–rutile direct junction. This new model clarifies the roles of anatase, rutile and the phase junction in achieving high photoactivity synergistically and provides the theoretical basis for the design of better photocatalysts by exploiting multi-phase junctions.

## Abstract 2

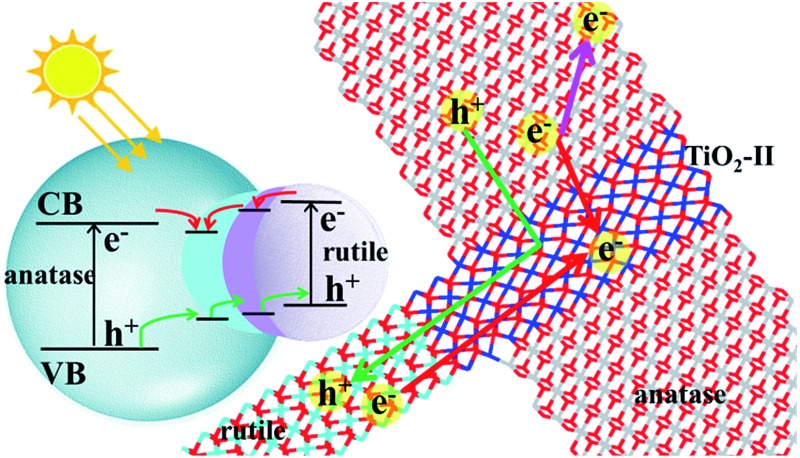
Theory resolves the anatase–rutile phase junction structure and characterizes its role in photocatalysis as a *single-way valve* modulating electron–hole separation.

## 1. Introduction

Searching for new materials that can efficiently harvest sunlight for energy conversion and chemical synthesis has been a great challenge in chemistry.^1^ TiO_2_, with a variety of stable phases, *e.g.* rutile, anatase and brookite,^2^ is found to exhibit tunable photoactivity towards different reactions depending on its structure and phases. Among the phases, rutile is shown to possess the highest photo-oxidation activity for oxygen evolution in water splitting,^3^ while anatase can have a higher activity in the photo-decomposition of organic molecules.^4^ More intriguingly, TiO_2_ composites with multiple phases, *e.g.* anatase–rutile, are able to work synergistically to yield the highest photoactivity for a wide range of reactions as first observed in the 1990s. To date, the mechanism of the enhanced photoactivity remains highly controversial; in particular, how the photogenerated holes and electrons migrate across phases must rank the top concern in the field.

The macroscopic morphology of anatase–rutile composites is shown to be complex, containing mixed structure units. Bickley *et al.*^5^ by using transmission electron microscopy (TEM) first observed an unusual microstructure in P25, a commercially available anatase–rutile composite catalyst, which contains anatase mostly in bulk covered by a thin overlayer of rutile. Later studies^6^ by the groups of Matsumura, Datye and Ying using TEM and diffuse reflectance spectroscopy showed that the rutile phase could also exist separately from the anatase particles in P25. With electron paramagnetic resonance spectroscopy (EPR), Gray’s group^7^ found small rutile crystallites interwoven with anatase crystallites in P25 and thus confirmed the presence of the interface. To reveal the physical origin of the enhanced photoactivity, some model biphase systems have been synthesized/constructed and tested for photoactivity. Using a sol–gel chemistry method, an anatase core/rutile shell structure was synthesized by Sung *et al.*^8^ and they found a higher rate for rhodamine B decomposition compared to other commercially available titania particles, including P25. Kawahara and coworkers^9^ prepared a patterned bilayer TiO_2_ photocatalyst film containing both anatase and rutile phases, which is shown to possess higher photocatalytic activity for CH_3_CHO oxidation than the individual phase. They found that the decrease of the patterning dimension helps to increase the photoactivity, which can then be correlated with the effective charge separation length. From these studies, the important role of the heterophase junction in photocatalysis is established: it can modulate the charge separation and may additionally act as the catalytic active site (“hot spot”) in reactions.^10^ Indeed, the charge separation mechanism is supported by recent studies on the band position of anatase and rutile.^11^ Using the hybrid QM/MM embedding technique and XPS measurements, Scanlon *et al.*^12^ calculated the ionization potentials of rutile and anatase (relative to the vacuum level) and determined that the conduction band minimum (CBM) of anatase is ∼0.4 eV lower than that of rutile.

However, questions remain on the microscopic structure and the direction of charge transfer across the heterophase junction. While no atomically resolved anatase–rutile phase junction has been reported experimentally, a series of structural models of the heterophase junctions has been proposed theoretically and their stability was also evaluated using empirical potential models based on molecular dynamics (MD) simulations^13^ or using first principles geometry optimizations.^14^ By matching the low-Miller-index anatase and rutile surfaces, Deskins *et al.* identified several interface structure models that are energetically likely, including (110)_R_//(101)_A_ and (100)_R_//(100)_A_ (the subscripts A and R indicate anatase and rutile phases, respectively).^13^ But, these optimized structures are generally rather disordered at the interfacial layers (∼4 Å) due to the large strain at the junction, which implies that the electron–hole transport through the interface is frustrated. These structural models are apparently contradictory with the high photocatalytic activity of mixed phase oxides.

Experimentally, the direction of photoelectron–hole migration has been controversial as well. The groups of Gray and Matsumura found that the photoelectrons can flow from rutile to anatase, which is caused by favorable thermodynamics of the lattice trapping sites on anatase.6c,^15^ By contrast, Nakajima and Komaguchi and their coworkers suggested that the photoelectrons flow from anatase to rutile while the holes transfer oppositely according to the measured band positions of anatase and rutile.^16^

The above theoretical and experimental results imply that the anatase–rutile phase junction might not be simple, *e.g. via* the epitaxial attachment of two phases. Indeed, using a novel stochastic surface walking (SSW) pathway sampling method,^17^ we recently found the lowest phase transition pathway between anatase and rutile crystals (using a 12-atom supercell).17*a* The SSW method is a method targeted for potential energy surface (PES) exploration, which was originally developed for aperiodic systems, *i.e.* molecules and clusters, and recently extended to crystal systems by coupling the degrees of freedom of the lattice with those of atoms.17*a* Unexpectedly, a high-pressure phase TiO_2_-II (α-PbO_2_-like form) is found to be the intermediate between rutile and anatase. The lowest energy pathway suggests a crystallographic correspondence between the three phases, *i.e.* rutile (101) plane being parallel with TiO_2_-II (001), (101)_R_//(001)_II_, and anatase(112) being parallel with TiO_2_-II (100), (100)_II_//(112)_A_.17*a* This finding is consistent with known experimental results on the appearance of the TiO_2_-II phase during the anatase to rutile transformation^18^ under lab synthesis conditions, and at the grain boundaries of rutile ores in nature.^19^ However, whether this intermediate phase is present at the anatase–rutile phase junction remains unknown. It is thus the goal of this work to analyse the likely phase junction structures and characterize their electronic properties to answer the puzzles on the photoactivity of the mixed oxides.

In this work we establish a new model for anatase–rutile phase junctions, namely a three-phase junction involving TiO_2_-II as the intermediate phase, by analyzing a number of possible phase junction structures, both direct two-phase junctions and indirect three-phase junctions. The structural information from the SSW solid-to-solid transition pathways is exploited to establish atomic models for the anatase–rutile junction. By optimizing the geometrical structure, computing the interfacial energy, the electronic band structure, the photon adsorption spectrum and comparing electron–hole energies at the phase junction, we propose a new mechanism for the charge separation between two phases and provide general guidance for optimizing the photoactivity of mixed oxides.

## 2. Calculation details

The density functional theory (DFT) calculations of mixed-phase TiO_2_ bulk were performed using SIESTA^20^ where optimized double-*ζ* plus polarization numerical atomic orbital basis sets^21^ were utilized along with the Troullier–Martins norm-conserving pseudopotentials.^22^ The exchange correlation functional utilized was at the generalized gradient approximation level of GGA-PBE.^23^ The energy cutoff for the real space grid used to represent the density was set as 250 Ry. The Broyden–Fletcher–Goldfarb–Shanno (L-BFGS) method was employed for geometry relaxation until the maximal force on each degree of freedom on an atom was less than 0.01 eV Å^–1^ and the stress was less than 0.5 GPa. The semi-core 3s and 3p states of Ti were included in all calculations. Monkhorst–Pack **k**-point sampling with 0.04 Å^–1^ spacing in the first Brillouin zone was utilized for the mixed oxide calculations (Table 2), which is verified to be enough to converge energetics, *e.g.* for a biphase crystal 3R/4II with a (***a*** = 4.41, ***b*** = 5.39, ***c*** = 17.23) cell, a (5 × 5 × 3) *k*-point mesh was utilized.

For comparison, the pure phase oxides^24^ of rutile (SG 136), anatase (SG 141) and TiO_2_-II (SG 60) are first optimized using DFT (see ESI Fig. S1†). The DFT optimized lattice parameters are (***a*** = 3.81, ***b*** = 3.81 and ***c*** = 9.68 Å) for anatase and (***a*** = 4.65, ***b*** = 4.65 and ***c*** = 2.97 Å) for rutile crystals, which agree well with the experimental lattice (anatase: ***a*** = 3.78, ***b*** = 3.78 and ***c*** = 9.49 Å; rutile: ***a*** = 4.59, ***b*** = 4.59 and ***c*** = 2.96 Å). There are two kinds of Ti–O bonds in both anatase and rutile bulk with the apical bonds (2.02 and 2.01 Å) about 2.5% longer than the equatorial Ti–O bonds (1.96 and 1.97 Å).^25^ In TiO_2_-II phase, an orthorhombic α-PbO_2_-like form, the optimized lattice parameters are ***a*** = 4.59, ***b*** = 5.60 and ***c*** = 4.93 Å, and the average Ti–O bond is 2.00 Å.

The post-GGA functional is essential to overcome the well-known delocalization error in pure LDA/GGA functionals, which underestimates the band gap and over-delocalizes the electron–hole states. To compute the band position more accurately for biphase crystals in Section 3.2, we also performed hybrid DFT calculations with the HSE06 functional^26^ using the CP2K/QUICKSTEP^27^ package based on the structure from PBE functional calculations, which is able to treat large oxide surface systems thanks to the auxiliary density matrix method^28^ for computing the Hartree–Fock exchange. In this method, the density matrix is re-expanded in a small auxiliary basis set, leading to massive speed-up of the Hartree–Fock exchange calculation. The cutoff of the real-space grid was set at 250 Ry. In the hybrid DFT calculations, the lattice of the crystal is generally multiplied to let each axis be larger than 10 Å and thus the **k**-point sampling was restricted to the gamma point only in the first Brillouin zone.

In Section 3.3, the energetics of the localized hole or electron in biphase crystals were calculated using spin-polarized DFT with the on-site Coulomb repulsion method, PBE + *U*, where the geometry of the oxide is fully relaxed to stabilize the localized charge carrier. The effective *U*–*J* terms, from linear response theory,^29^ were 3.5 eV on Ti 3d orbital for electrons, or 10 eV on O 2p for holes. These calculations were performed in the plane wave and ultrasoft pseudopotential^30^ framework as implemented in the QUANTUM-ESPRESSO package.^31^ The kinetic energy cutoffs of 30 and 300 Ry were chosen for the wave functions and augmented charge densities, respectively. The BFGS method was employed for geometry relaxations until the maximal forces on each relaxed atom were less than 0.001 Ry/Bohr. A (3 × 3 × 1) Monkhorst–Pack *k*-point mesh was utilized for energy and structural calculations.

The DFT + *U* approach for computing localized holes was first introduced by Deskins and Dupuis,^32^ whose calculation results show reasonable agreement with the available experimental data. The value of *U* (10 eV) used here is determined from a first principles linear-response approach,^29^ which has been utilized for 3d materials with good accuracy comparable with CCSD(T) calculations.^33^ In this work, our calculated band edge difference from the DFT + *U*, *i.e.* VBM difference of anatase and rutile is ∼0.30 eV (see Table 3), is also consistent with those from previous experimental and theoretical studies.^12^

## 3. Results

### 3.1 Structure of heterophase junction

#### (a) Identifying coherent phase junctions with low strains

We start by exploring the likely form of the anatase–rutile phase junctions. Our recent work using the SSW pathway sampling method17*a* shows that the presence of an intermediate high-pressure phase TiO_2_-II can significantly reduce the phase transition barrier between rutile and anatase. Note that the phase transition pathways from SSW sampling, although they do not directly tell the phase junction structure, they do provide critical information on the crystallography correspondence, namely, the orientation relation (OR), related to the lowest energy pathways.

Based on the knowledge from SSW sampling pathways, we selected anatase(112), rutile(101) and TiO_2_-II(100) and TiO_2_-II(001) as the main candidates for the interfacial planes constituting the phase junction, since these crystallography planes dominate the orientation relation in the lowest energy pathways. In Table 1 and also in Table S1,† we first compared our junction models with previously studied models, both the direct two-phase junction and the indirect three-phase junction. The interfacial strain energy can be computed by using the matrix algebra in finite strain theory of mechanics^34^ as detailed below.

**Table 1:** Models for anatase–rutile phase junction, the computed strain energy and the interfacial energies *γ*_int_ (J m^–2^). The phase junction is constructed by joining two phases (P1 and P2) according to the orientation relation (OR)^*a*^

OR	P1		P2		*S*	*γ*_*i*nt_
	***a*** (Å)	***b*** (Å)	***a*** (Å)	***b*** (Å)		
**Direct Model**						
**I**	5.38	5.54	5.51	4.65	2.37	0.58
**II**	5.51	5.51	5.34	3.81	3.83	0.88^*b*^
**III**	5.34	3.81	4.65	4.65	2.49	—
**IV**	9.68	3.81	6.57	2.97	3.81	—

**Indirect Model**						
**V**	5.51	4.65	5.59	4.60	2.01	0.01
**VI**	5.59	4.95	5.38	5.54	2.18	0.11
**VII**	5.59	6.76	5.38	5.54	2.57	—
**VIII**	5.38	5.54	5.59	4.60	2.35	—

^*a*^The OR includes a pair of parallel crystallography planes (*hkl*)_P1_//(*hkl*)_P2_ that are attached to each other (with lattice parameters *a* and *b*) and a pair of parallel directions [*uvw*]_P1_//[*uvw*]_P2_. Here OR are as follows. **I**: (112)_A_//(101)_R_, [110]_A_//[101]_R_; **II**: (111)_R_//(101)_A_, [011]_R_//[010]_A_; **III**: (101)_A_//(001)_R_, [010]_A_//[010]_R_; **IV**: (100)_A_//(110)_R_, [001]_A_//[110]_R_; **V**: (101)_R_//(001)_II_, [101]_R_//[100]_II_; **VI**: (100)_II_//(112)_A_, [010]_II_//[110]_A_; **VII**: (101)_II_//(112)_A_, [101]_II_//[110]_A_; **VIII**: (112)_A_//(001)_II_, [110]_A_//[100]_II_.

^*b*^From ref. 35.

Let us define two lattices as **T** and **M**, both (3 × 3) matrixes of a lattice. A deformation gradient *F* matrix transforms an initial lattice *T* to a final lattice *M*, as

1 **FT** = **RBT** = **M**

2 **F** = **RB**

where **R** is a rigid-body rotation matrix and **B** is a lattice deformation matrix, representing the generalized Bain deformation. The Cauchy–Green deformation tensor is

3 **C** = **F**^**T**^**F** = (**T**^**T**^)^**–1**^**M**^**T**^**MT**^**–1**^

where **C** is rotational invariant. The principal axes are the eigenvectors (*e*_*i*_, *i* = 1,2,3) of the Cauchy–Green deformation tensor

4 **Ce**_***i***_ = ***l**_**i**_***e***_**i**_*

The strain energy of the lattice deformation is defined the sum of three eigenvalues, *l*_*i*_

5 *I* = tr(**F**^**T**^**F**) = **l**_**1**_ + **l**_**2**_ + **l**_**3**_

For the strain at the interface, we are dealing with a two-dimensional problem instead of a three-dimensional crystal deformation. Therefore, we can define the strain energy at the interface similarly as

6 *S* = **l**_**1**_ + **l**_**2**_

where **l**_**1**_ and **l**_**2**_ are the eigenvalues measuring the strain along the principal axis of the interfacial plane. The magnitude of *S* is related to the definition (order) of lattices **T** and **M**, and thus for comparison of different junctions, we define *S* as being equal to or larger than 2 (*e.g.* volume expansion from **T** to **M**). Obviously, the closer to 2 the *S* value is, the lower the strain energy.

From Table 1 and ESI Table S1,† we found that the strain energy *S* for OR **I**, the direct interface (101)_R_ and (112)_A_ is among the smallest, *i.e.*, 2.37, for the direct interface models (the equivalent density condition of two phases is enforced in searching for the best matched interface). More importantly, we found that by introducing the intermediate TiO_2_-II phase, thus the indirect junction, the strain energy at the interface can be reduced to 2.01 and 2.18 for rutile/TiO_2_-II and anatase/TiO_2_-II interfaces in OR **V** and **VI**, respectively. These OR pairs are indeed those obtained from the lowest energy pathways in SSW pathway sampling. In the following, we will construct the phase junction structure using these low strain candidates and compare their interfacial energies from first principles.

#### (b) TiO_2_-II as the intermediate phase between rutile and anatase

**Fig. 1:**
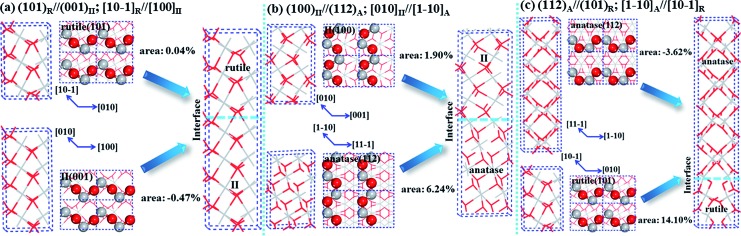
Construction of biphase phase-junctions using OR. (a) Rutile/TiO_2_-II (OR **V**), (b) TiO_2_-II/anatase (OR **VI**) and (c) anatase–rutile (OR I), Ti: grey; O: red.

Based on the ORs in Table 1, it is feasible to construct the heterophase junctions for rutile/TiO_2_-II and anatase/TiO_2_-II with the lowest strain energy (OR **V** and **VI**). We investigated the rutile/TiO_2_-II phase junction with four different ratios, namely, 3R/4II (3 layer rutile and 4 layer TiO_2_-II per cell), 3R/2II, 4R/1II, 6R/1II, and similarly the anatase/TiO_2_-II phase junction with four different ratios, namely, 4A/4II (4 layer anatase and 4 layer II per cell), 8A/4II, 8A/2II, 12A/2II. These biphase super-lattice crystals can be constructed as illustrated in Fig. 1a, and both the lattice and atom coordinates can then be fully optimized using DFT.17*a* We have compared in Table 2 the shortest Ti–O bonds (apical and equatorial bonds of TiO_6_ octahedron) in the biphase crystals, inside the parent phases (P1 and P2) and at the interface layers.

**Table 2:** Energetics of the biphase crystals at different phase ratios together with the optimized lattice parameters (*a* and *b*) and the Ti–O bond (*d*, Å) lengths

P1/P2^*a*^	Δ*E*^*b*^ (eV)	*γ*_int_ ^*b*^ (J m^–2^)	***a*** (Å)	***b*** (Å)	P1^*c*^	Inter	P2
**Rutile/TiO**_**2**_ **-II (OR V)**							
3R/4II	0.01	0.02	4.61	5.57	1.92(1.95)	2.00	2.00
3R/2II	0.01	0.03	4.60	5.56	1.93(1.97)	1.98	2.00
4R/1II	0.00	0.01	4.61	5.57	1.95(1.97)	2.00	—
6R/1II	0.01	0.03	4.61	5.57	1.96(1.96)	1.99	—

**Anatase/TiO**_**2**_ **-II (OR VI)**							
4A/4II	0.04	0.19	5.54	5.13	1.91(1.97)	1.99	2.00
8A/4II	0.04	0.28	5.50	5.25	1.92(1.99)	1.99	2.01
8A/2II	0.03	0.14	5.45	5.40	1.94(2.00)	2.00	2.02
12A/2II	0.02	0.11	5.43	5.47	1.89(1.96)	2.00	2.03

**Rutile/anatase (OR I)**							
8A/15R	0.11	1.49	4.86	5.52	1.91(2.00)	1.99	1.89(1.98)
16A/15R	0.12	2.19	5.06	5.51	1.94(1.98)	1.97	1.90(1.91)
8A/3R	0.11	0.65	5.23	5.60	1.91(2.02)	1.93	1.84(2.08)
12A/3R	0.07	0.58	5.29	5.62	1.93(2.01)	1.93	1.85(2.05)

^*a*^The name and ratio of two phases, *e.g.* 3R/4II stands for 3-layer rutile and 4-layer TiO_2_-II to form a biphase crystal.

^*b*^Δ*E*, see eqn (7) and *γ*_int_, see eqn (8).

^*c*^For anatase and rutile, there are two types of Ti–O bonds in bulk TiO_4_ octahedron: equatorial and apical Ti–O bonds.

For the rutile/TiO_2_-II phase junction, the DFT optimized lattice parameters along the ***a*** axis at [101]_R_ and the ***b*** axis at [010]_R_ are 4.61 × 5.57 Å, being very similar to 4.63 × 5.55 Å for rutile and 4.61 × 5.60 Å for TiO_2_-II (see Fig. 1a). In 3R/4II (Fig. 1a), rutile (101) expands by 0.04% in area and TiO_2_-II (001) shrinks by 0.47%. As a result, the Ti–O bonds at the rutile side of the biphase crystal (∼2.06 Å) are slightly longer than that of the bulk rutile phase (∼2.01 Å) due to the presence of the TiO_2_-II phase. All Ti atoms are six-fold coordinated (Ti_6c_) with neighboring O atoms, and all O atoms are three-fold coordinated (O_3c_) with all three Ti–O bonds in the same plane. Apparently, the Ti and O bonding environments at the rutile/TiO_2_-II phase junction are similar to those in the bulk parent crystals, indicating a favorable structure match at the atomic level.

For the anatase/TiO_2_-II phase junction, the lattices of the two phases are not as well matched as those of rutile and TiO_2_-II because the density of anatase (3.78 g cm^–3^) is much lower than that of TiO_2_-II (4.14 g cm^–3^). In the optimized 4A/4II biphase crystal, the lattice along ***a*** at [110]_A_ and ***b*** at [001]_A_ is 5.54 × 5.13. By comparing with these lattice parameters in anatase bulk (5.35 × 5.64 Å) and TiO_2_-II bulk (5.62 × 4.97 Å), one can see that the surface area of TiO_2_-II(100) increases by 1.90% while that of anatase(112) decreases by 6.24% in forming the phase junction, indicating a large strain developed on the anatase side. With the increase of anatase ratio in the super-lattice, the strain on the anatase side can be gradually released. For example, in 12A/2II the surface area of anatase(112) only decreases by 1.81%. Hence, thin TiO_2_-II layers are preferred at the interface of the biphase crystal. In the biphase crystal the Ti and O atoms remain as Ti_6c_ and O_3c_ as in the parent phases; the Ti–O bond length is maximally shortened by 0.07 Å which occurs in the anatase parts (∼1.89 Å); and the Ti–O bond length at the interface is in between those of anatase and TiO_2_-II, reflecting the transition nature from one phase to another.

#### (c) Direct phase junction between rutile and anatase

We also examined the direct interface by joining anatase(112) with rutile(101) (OR **I** in Table 1) at different phase ratios, namely, 8A/15R, 16A/15R, 8A/3R and 12A/3R. The procedure to construct these phase junctions is similar to that described above for other biphase crystals. The results are also listed in Table 2. We note that the previous theoretical work has analyzed some direct interface models, involving low Miller index surfaces, such as (110)_R_//(101)_A_, (110)_R_//(100)_A_, (100)_R_//(101)_A_ and (001)_R_//(101)_A_.^13^ But anatase(112) and rutile(101) in forming a phase junction was not considered previously. The anatase(112) surface is known to be important for initiating the phase transition from experiment^36^ and from our recent SSW pathway sampling.^17^

In Fig. 1c, we illustrate the construction procedure for the anatase–rutile biphase crystal with the ratio of 8A/3R. For the optimized 8A/3R biphase crystal, the surface area of (101)_R_ needs to increase by 14.10% while that of (112)_A_ shrinks by 3.62% with respect to the their corresponding pure phase. To be specific, the ***a*** axis along [101]_R_ changes from 5.55 Å in rutile and 5.38 Å in anatase to 5.23 Å in the biphase crystal, and the ***b*** axis along [010]_R_ changes from 4.63 Å in rutile and 5.65 Å in anatase to 5.60 Å in the biphase crystal. With the increase of the anatase ratio (Table 2), the lattice parameters ***a*** and ***b*** in the biphase crystals increase gradually, approaching the bulk values of anatase (***a*** = 5.38, ***b*** = 5.65 Å). At the interface, a large distortion of the bonding of Ti and O occurs, mostly in the rutile part due to its larger lattice expansion. As a result, the Ti–O bond length increases by up to 0.27 Å (from 2.02 Å in the bulk to 2.29 Å at the interface); and the interface O and Ti are two (O_2c_) and five coordinated (Ti_5c_), respectively, which are one less than those in the bulk. It is noticed that Ti_5c_ and O_2c_ are common on TiO_2_ surfaces, *e.g.* rutile(110). This indicates the direct interface of the anatase–rutile biphase crystal cannot fully accommodate the dangling bonds at the phase boundary.

#### (d) Interfacial energy of phase junction

To evaluate the stability of the biphase crystals at different phase ratios, we have computed the energy cost (Δ*E*) of forming the biphase crystals with respect to the pure bulk phases and the interfacial energy (*γ*_int_) of the phase junction:7
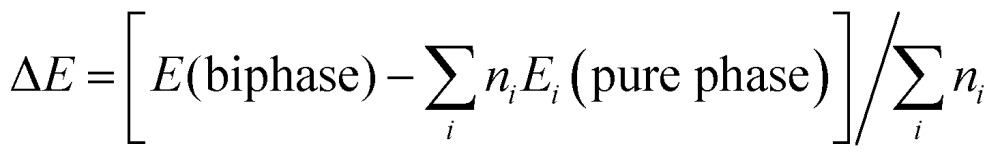

8
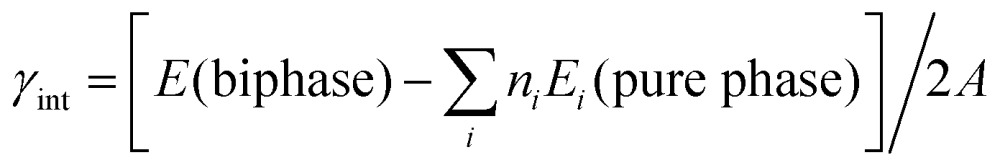
where *E*(biphase) is the total energy of the biphase crystal, *E*_*i*_(pure phase) is the energy of the pure phase, *n*_*i*_ is the number of TiO_2_ units of the different phase components in the biphase and *A* is the surface area of the interface. Obviously, the lower *γ*_int_ is, the more stable the interface will be.

Using eqn (8), *γ*_int_ for different ratio biphase R/II and A/II crystals have been calculated and are listed in Table 2. For R/II composite, the 4R/1II biphase crystal is the most stable with a low *γ*_int_ of 0.01 J m^–2^. For A/II composite, the 12A/2II biphase crystal has the lowest *γ*_int_, 0.11 J m^–2^, which is larger than that in 4R/1II. By contrast, the calculated *γ*_int_ for the direct anatase–rutile junction is 0.58 J m^–2^ (this corresponds to a strain ∼16%, see Table 1) which is lower than the reported *γ*_int_ of anatase–rutile between (111)_R_ and (101)_A_ (0.88 J m^–2^).^35^ In fact, *γ*_int_ of 0.58 J m^–2^ is already quite large compared to the common magnitude of surface energies and we regard 16% strain as the general upper limit of strain for TiO_2_ interfaces. Indeed, the *γ*_int_ of the direct model is significantly larger than the TiO_2_-II involved indirect models. These DFT calculated energetics are consistent with the structural features from strain analysis in Table 1: in general the large strain yields the unstable interface.

It is noted that the superlattices with very thin layer TiO_2_-II may not be realistic models for the anatase–rutile junction and therefore the interfacial energies therein are more of theoretical interest. Nevertheless, from the energetics of these ideal models, we can identify the general trend on the stability of the interface, which demonstrates that with the reduction of the layer of TiO_2_-II phase, the strain developed at the interface can be further reduced and the interface becomes more stable.

From both the geometrical structure and the interfacial energetics, it is clear that the presence of TiO_2_-II as an intermediate phase in between rutile and anatase can be essential to stabilize the anatase–rutile phase junction. The rutile side interface (R/II) is facile to form with low *γ*_int_, whilst the anatase side interface (A/II) dominates the energy cost to grow the phase junction and the fraction of TiO_2_-II is expected to be low to minimize the A/II interfacial energy. The phase transition rate-determining step is thus expected to relate to the transformation of anatase to the intermediate TiO_2_-II phase. This could have some profound implications for the structure and photocatalytic behavior of anatase–rutile nanoparticles, *e.g.* a narrow interface region with the close atomic contact between phases, the critical size of anatase particle and the ratio between rutile and anatase for high photoactivity. We will return to discuss these in more detail in Section 4. In the following, we will focus mainly on the indirect interface models to understand the physicochemical properties of the unusual three-phase junction.

### 3.2 Electronic structure of mixed phases

**Fig. 2:**
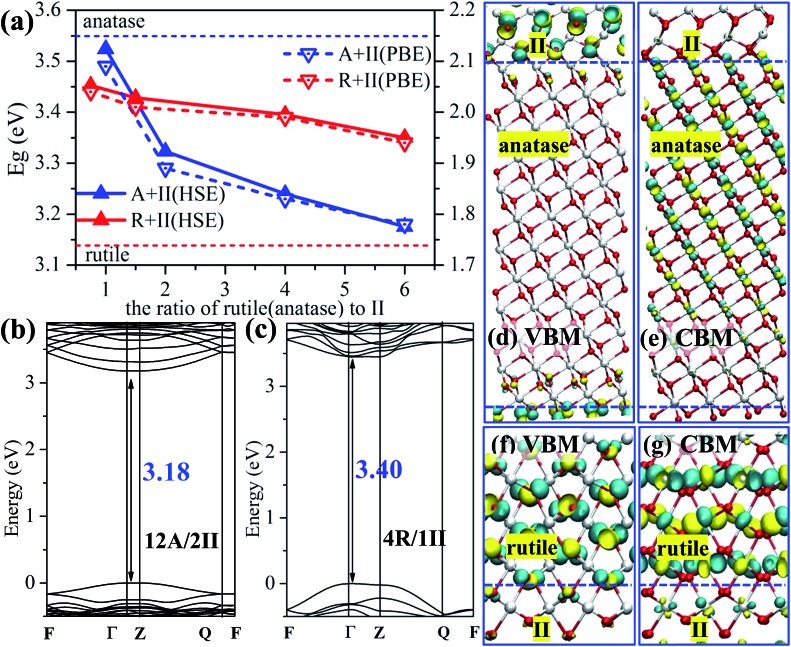
Electronic structure of anatase/TiO_2_-II/rutile three-phase junction. (a) The band gap for rutile/TiO_2_-II and anatase/TiO_2_-II biphase crystals as calculated by HSE06 (left *y*-axis, solid lines) and PBE (right *y*-axis, dotted lines) functionals. Red: rutile; blue: anatase. (b, c) The band structures for the biphase crystals 12A/2II and 4R/1II. (d–g) The 3D isosurface contour plots of the VBM and CBM using the HSE06 functional for 12A/2II and 4R/1II biphase crystals. White: Ti atoms; red: O, the isosurface value is set as ±0.14 e Å^–3^.

The band gap is a key property related to the optical absorption behavior of a photocatalyst. Using the superlattice structure presented above, we have computed the band gap values of the pure phases and the mixed phase oxide crystals using DFT with both the PBE functional and the hybrid HSE06 functional. The results are summarized in Fig. 2a. Our results show the PBE band gap for different ratio mixed phase crystals yields the same trend as the results from HSE06, except that the predicted PBE band gap is systematically narrower compared to that of HSE06. This is not surprising as the pure DFT functional tends to underestimate the band gap due to the lack of the exact exchange. The HSE06 band gap is in general more consistent with experimental values for the pure phases, *e.g.* rutile: 3.03 eV from experiment^12^ and 3.13 eV from HSE06; anatase: 3.20 eV from experiment^37^ and 3.55 eV from HSE06.

Fig. 2a shows that the band gaps of rutile/TiO_2_-II crystals are rather constant at different ratios, *i.e.* 3.45, 3.43, 3.40 and 3.35 eV from HSE06 functional for R/II ratios of 0.75, 1.5, 4 and 6, respectively. By contrast, the band gap of the anatase/TiO_2_-II composite drops rapidly from 3.52 to 3.18 eV with the increase of the A/II ratio from 1 to 6. The 12A/2II biphase crystal has a band gap of 0.37 eV lower than pure anatase. These results imply that the presence of TiO_2_-II significantly modifies the electronic structure of anatase but it has little effect on rutile.

To provide a better understanding of the electronic states of the mixed phase, we chose the two most stable biphase crystals, 12A/2II and 4R/1II (see Table 2), and plotted the electronic band structure using the optimized superlattice structure, as shown in Fig. 2b and c (this is carried out using DFT PBE calculations and a band gap offset, *i.e.* a scissors operator, is introduced to shift up all the unoccupied states using the band gap difference between PBE and HSE06 results^38^). The reciprocal-space fractional coordinates for the *k*-points used are the following: *Γ* = (0, 0, 0), *F* = (0, 0.5, 0), *Q* = (0, 0.5, 0.5) and *Z* = (0, 0, 0.5).

The 12A/2II biphase crystal exhibits a direct band gap of 3.18 eV located at the *Γ* point (because of the large *z*-axis in the superlattice, 33.06 Å, the gap at the *Z* point is identical to that at the *Γ* point). The topmost (valence band maximum, VBM) and the lowest (conduction band minimum, CBM) bands of the biphase (12A/2II) are non-degenerate at the *Γ* point with a dispersive character, indicating a delocalized behavior of the wavefunction, which is desirable for the transport of photogenerated electrons and holes. By comparing with the band structure of pure anatase, we found that the characteristics of the VBM and the CBM in 12A/2II are rather different from those of bulk anatase and bulk TiO_2_-II. Indeed, by plotting the wavefunction in Fig. 2d and e, we found that the VBM is mainly located at the TiO_2_-II side around the lattice O sites, while the CBM is mainly distributed at the anatase side around the lattice Ti sites. Such a significant spatial separation of the VBM and CBM can effectively stabilize the photogenerated electron and holes after the charge separation.

For 4R/1II, it has a direct band gap of 3.40 eV at the *Γ* point (Fig. 2c). The topmost two branches of VB are degenerate at the *Q* point and nondegenerate at the *Γ* point, while the lowest two branches of CB are degenerate at the *Γ* point. These characteristics are similar to those of bulk rutile, showing that the electronic structure of biphase 4R/1II retains the features of rutile. Consistently, from the plotted wavefunction distributions shown in Fig. 2f and g, we found that both VBM and CBM are mainly located in the rutile phase, which is different from the 12A/2II biphase crystal. It suggests that the separation of the charge carriers is not likely at the TiO_2_-II/rutile junction. Overall, the electronic structure at the phase junction can be rather different from anatase and rutile, particularly at the anatase-side interface where the VBM and CBM can be spatially separated.

Next, it is of interest to further compare the optical properties of the A/II biphase crystal with those of the pure anatase phase, which has great importance in photocatalysis. Since the anatase-side interface exhibits interesting band structure features, we calculated the optical absorption spectra of the biphase 12A/2II and compared it with the pure anatase phase, where the complex dielectric function needs to be evaluated.^39^

**Fig. 3:**
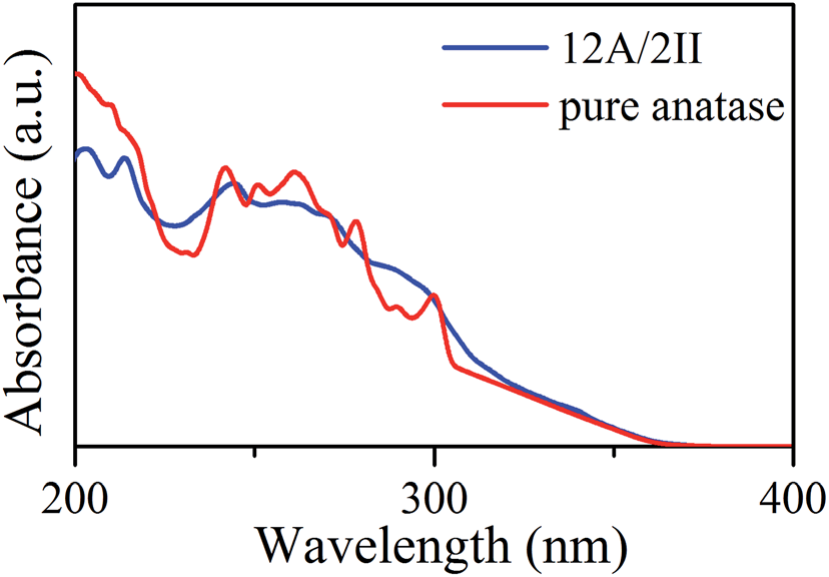
Optical absorption coefficient spectra for the 12A/2II biphase and pure anatase.

Interestingly, the presence of the heterophase junction has little effect on the optical adsorption spectra. As shown in Fig. 3, we found that the absorption of both biphase 12A/2II and pure anatase starts from ∼378 nm, corresponding to a similar optical band gap of 3.28 eV (exp. value for anatase is 3.2–3.3 eV).^40^ Obviously, the photon adsorption in both cases should mainly proceed on the anatase phase. This could be understood considering that the spatial overlap of VBM and CBM in the 12A/2II crystal is negligible and thus the direct excitation of electrons from VBM to CBM is unlikely. The previous experimental work has measured the optical band gap of mixed phase P25 using UV-vis diffuse reflectance spectra and found that the optical band gap of P25 is 3.14 eV, only 0.08 eV lower than that of pure anatase.^41^ This is consistent with current theoretical results. It is thus concluded that the role of the interface is to facilitate the charge separation during the carrier relaxation and transport, instead of enhancing the optical adsorption.

### 3.3 Thermodynamics of electron and hole across the phase junction

The above band structure results are obtained from the static geometrical structures of biphase crystals. In reality, when the charge migrates across the solid, the local geometrical structure can relax to help stabilize the charge carriers. This could yield unexpected new charge trapping sites and therefore is critical in photocatalysis. To further unravel the thermodynamics of charge transfer in the mixed-phase TiO_2_, we utilize the post-GGA functionals, PBE + *U*, to investigate the localized charge migration at and across the phase junction. With PBE + *U*, the added hole–electron can localize on one single atom at different positions of the crystal corresponding to variant electronic configurations, which allows us to evaluate the thermodynamics of the hole–electron across the phase junction.

In this work, we consider the addition of an excess electron and hole separately in calculations, the so-called individual polaron, where the possible excitonic/local field is neglected. In this way, a compensating uniform background charge is introduced to re-establish the neutrality of the supercell. Concerning to the possible excitonic/local field effect, we refer to the previous work by Valentin and Selloni, who utilized the hybrid B3LYP functional^42^ for the anatase system and found that the trapping energy for an exciton is in fact less than the sum of the trapping energies for two individual polarons. They suggest that an exciton prefers to split into two individual polarons thermodynamically.

**Fig. 4:**
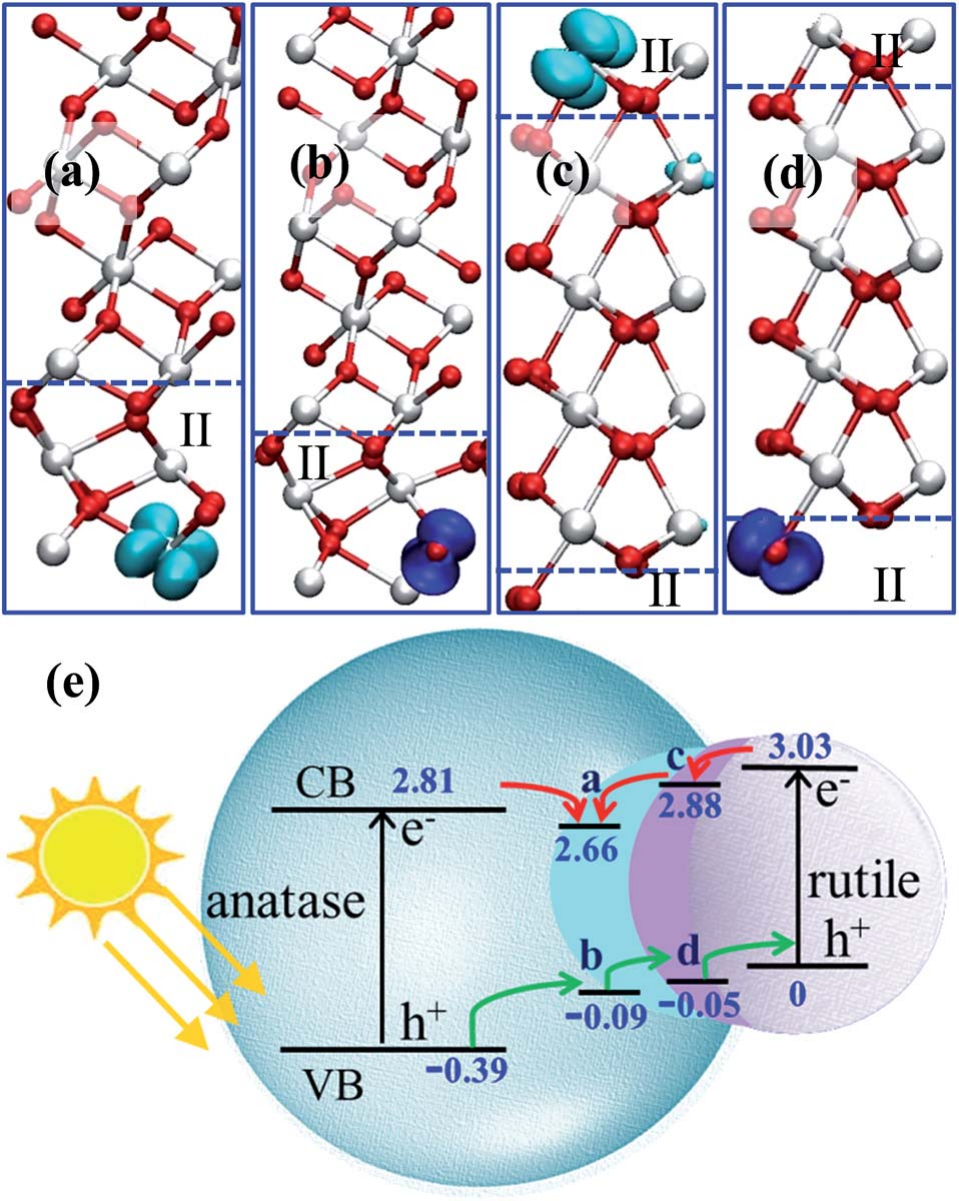
(a–d) 3D contour plots of the spin density of the electron and hole in the A/II interface and in the R/II interface, showing the characteristic d-like and p-like distribution on a single Ti/O atom. The isosurface value is set as 0.04 e Å^–3^. (e) The relative band levels of the three-phase junction in anatase–rutile mixed phase photocatalysts using the data in Table 3 (the energy of the hole in rutile is set as zero for reference). The relative band positions and band gaps for anatase and rutile are adopted from a recent experiment.^12^

By plotting the spatial distribution of the spin density corresponding to the added electron and hole (as shown in Fig. 4a–d), we found that the extra electron in anatase tends to delocalize throughout the whole anatase phase, while in other calculations, the extra electron or hole can be localize on a single Ti or O site. This delocalization of the electron in anatase bulk implies fast electron migration within anatase, which is consistent with the experimental finding that electron transfer in anatase is 2 orders faster than that in rutile.^43^

We noticed that the localized extra electron has introduced a large local structural relaxation. For example, for the electron at the A/II interface, the local Ti–O bond has been significantly lengthened by 0.43 Å, while the Ti–O bond is only lengthened by 0.01 Å when the electron is in the anatase bulk. Therefore, the computed energetics of the charge carriers already reflects the local structural response in stabilizing the extra charge.

By calculating the energy difference for the hole–electron at different positions of the three-phase junction, we found that the hole at the TiO_2_-II side is 0.30 eV more stable than that at the anatase side, and 0.05 eV less stable than that at the rutile side at the R/II junction, as listed in Table 3. The hole transfers from anatase to rutile is therefore facile without barrier. By contrast, the interfaces at the three-phase junction will act as the trapping sites for photogenerated electrons and the electron transfer from rutile to anatase is hindered by at least 0.15 eV barrier based on the thermodynamics.

**Table 3:** The relative stabilities (eV) of hole and electron at the three-phase junction and the rutile–anatase direct junction^*a*^

	Inter.	Bulk anatase	Bulk rutile
**Indirect Model**			
Hole	0	0.30	–0.05
Electron	0	0.15	0.15

**Direct Model**			
Hole	0	0.42	0.11
Electron	0	–0.04	–0.16

^*a*^The three-phase junction is represented by the anatase/TiO_2_-II crystal with a 12A/2II model and the rutile/TiO_2_-II crystal with a 4R/II model. The rutile–anatase direct junction is represented by a 8A/15R model. In each calculation, the energy of hole–electron at the interface (Inter.) is set as reference.

By contrast, we found interestingly that the direct anatase–rutile phase junction is in fact not able to promote the electron and hole separation (see Table 3 bottom panel). The hole is more stable at the rutile phase compared to at the anatase phase by 0.31 eV, while the electron slightly prefers the rutile than the anatase phase. This energy difference of the hole (0.31 eV) is in good agreement with those (∼0.39 eV)^12^ deduced from the band levels in the static band structure calculations and the experimental values. The electron stability is different from those reported previously and we attribute this to the local structural relaxation in the biphase crystal. Importantly, the direct interface between anatase and rutile provides a trapping site for the hole and at the same time hinders the electron transfer. The behavior of the two-phase junction is distinct from that of the three-phase junction, indicating that the phase junction structure has a great influence on the electron and hole migration.

Using the calculated energetics of charge carriers in the three-phase junction, we can schematically summarize the overall band alignment of the three-phase junction in Fig. 4e. Note that the relative band positions and band gaps for anatase and rutile are adopted from the recent experiment,^12^ which shows that the band position of CBM of anatase is lower than rutile by 0.22 eV and VBM of anatase is lower than rutile by 0.39 eV. Fig. 4e shows that: (i) the photogenerated hole can transfer facilely from anatase to rutile assisted by the thin TiO_2_-II-based junction; (ii) the electron trapping sites are present at the three-phase junction and thus the photoelectron migration from rutile to anatase is thermodynamically hindered.

## 4. Discussion

### 4.1 3-D model of phase junction and experimental evidence

**Fig. 5:**
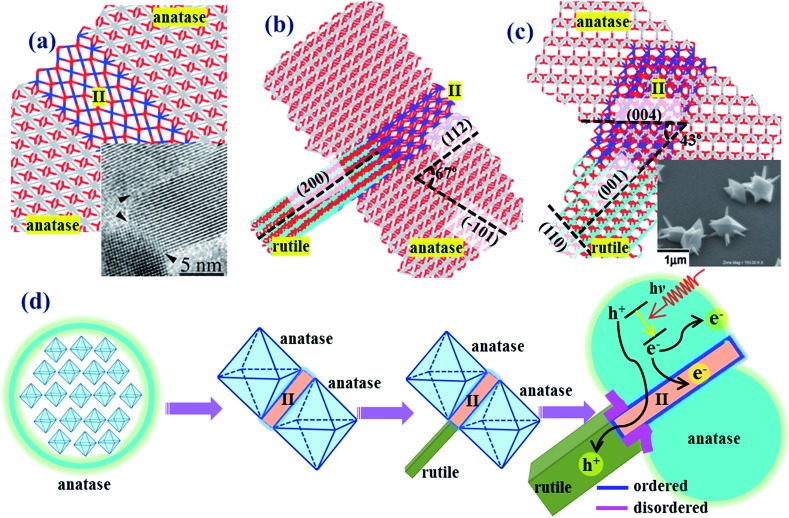
Theoretical atomic models for (a) anatase(112)-twin junction and (b and c) the anatase–rutile phase junction involving the intermediate phase of TiO_2_-II. The dihedral angle of the planes is denoted in the picture. For clarity, the Ti atoms in anatase, rutile and TiO_2_-II are represented by gray, blue and cyan, respectively. The inset in (a) shows the HRTEM patterns of anatase(112)-twin from ref. 44 and that in (c) from ref. 45. (d) A scheme showing the macroscopic structure evolution picture for anatase–rutile mixed oxide, containing anatase, rutile and TiO_2_-II. The concentration of TiO_2_-II is exaggerated to show clearly the spatial relation of the three phases.

To illustrate the indirect phase junction model of anatase–rutile in real space, we have constructed an atomistic model of the anatase–rutile junction involving the TiO_2_-II as the intermediate phase, as shown in Fig. 5, using the OR determined theoretically, *i.e.* (112)_A_//(100)_II_ and (101)_R_//(001)_II_.17*a*Fig. 5a shows the anatase twin structure with anatase(112) as the interface, in which a thin 4-layer of TiO_2_-II is present as the intermediate. The rutile phase can further attach to the anatase/II twin structure *via* the TiO_2_-II phase, and the R/II junction is thus rotated by 90 degrees with respect to the A/II junction. The final constructed atomic model is as shown in Fig. 5b and c from two different side views.

The macroscopic structure evolution of anatase–rutile mixed oxide can thus be deduced as shown in Fig. 5d, containing anatase and rutile and the intermediate phase TiO_2_-II. The three-phase phase junction has two propagation directions, perpendicular to anatase(112) and to rutile(101), which forms a shallow channel for hole transport. With the increase of the rutile and anatase particle size, the three-phase junctions are buried inside the nanoparticles and the direct interface between rutile and anatase will start to form by joining the neighboring particles. These direct phase junctions are present together with the structurally ordered three-phase interface, yielding a complex nature of the phase junction between rutile and anatase.

Now we turn to the experimental evidence on the anatase–rutile phase junction. In fact, the stepped anatase(112) is long known to be a key facet to initiate the phase transformation as observed from experiment and theoretical modelling.^36^ Penn’s group^44^ found that by using anatase particles as a seed, the attachment of separated particles occurs mainly *via* the (112) and (001) surfaces, which is the initial step towards the phase transformation from anatase to rutile. Using high-resolution TEM, they even observed the atomic structure of the anatase(112)-twin junction and a few layers of a newly-emerged phase (they called that a brookite-like phase) freezing in between the (112)-twin junction. In Fig. 5a insert, we also compare the experimentally observed atomic structure of the anatase(112)-twin junction from HRTEM^44^ with our theoretical model in Fig. 5a.

As for the rutile–anatase phase junction, there is no atomic level evidence for the presence of a structurally ordered phase junction. A recent experiment by Hosono *et al.*^45^ has synthesized rutile–anatase biphase crystals under hydrothermal conditions. They found that the rutile crystals can grow as a nanopin-like structure on a micro-anatase octahedral single crystal. Such a structure as observed in SEM is shown in the insert of Fig. 5c. It shows that the area of the anatase–rutile junction is low in concentration compared to the exposed rutile and anatase facets. In other words, a direct contact between anatase and rutile crystals is thermodynamically unstable compared to the exposed crystal facets of rutile and anatase. These facts are consistent with our proposed 3-D phase junction in Fig. 5.

### 4.2 New mechanism of electron–hole separation and charge transport

Finally, we are at the position to discuss the implications of our three-phase junction model in the context of photocatalysis. The synergistic effect of the anatase–rutile mixed phase in photocatalysis^46^ has been often explained as a consequence of enhanced charge separation by the phase junction^43^ with the view that a close contact between anatase and rutile phases is the prerequisite^47^ for a high photoactivity. The relative band positions of anatase and rutile also appear to support this mechanism, in which the phase junction acts as a modulating gate to separate photogenerated electrons and holes. The holes tend to accumulate on the rutile and the electrons prefer the anatase phase, namely, a dual-way valve model for charge separation. The other mechanisms are more sophisticated and are less recognized.^48^ For example, the band gap variation mechanism^49^ suggests that the band gap of mixed phase oxides may vary as a function of the particle size distribution and the rutile phase content. However, our previous theoretical work^50^ showed that the band gap of TiO_2_ nanoparticles converges quickly to their bulk values. For nanoparticles above 2.5 nm, the band gap is little affected by the particle size.

The dual-way valve model for charge separation however met some difficulties in rationalizing important experimental findings in photocatalysis. Because the structurally ordered interface for anatase–rutile was not confirmed by theoretic simulation or experimental observation previously, it remains highly controversial whether both electrons and holes can migrate across the phase junction without being trapped. It was also found that the best photoactivity for water oxidation in anatase–rutile composite catalyst requires generally a much higher concentration of anatase than that of rutile, *i.e.* rutile–anatase being ∼20 : 80,^51^ which is not a more straightforward 50 : 50 ratio as would be expected from the dual-way valve model. The low ratio for rutile is quite surprising considering that rutile is found to be more active than anatase for oxygen evolution and naphthalene oxidation.15*c* Finally, based on the dual-way valve model, it is expected that the holes and electrons are very likely to recombine at the phase junction, which should in turn limit the photoactivity.

Instead, our current results support a single-way valve model based on a structurally ordered three-phase junction. At the three-phase junction, the migration rates for electrons and holes across the phase junction differ significantly. According to the calculated energetics for localized holes and electrons, we found that only holes can travel facilely from anatase to the interface region and to rutile, *i.e.* a single-way valve for hole transfer. The electron migration is slowed by the trapping site at the A/II interface, which could stabilize photoelectrons by 0.15 eV compared to in anatase bulk. From microkinetics, this indicates that the electron migration across the junction is about three orders of magnitude slower compared to that of holes at the ambient temperature.

While the three-phase junction can act as an electron trapping site, we expect that the electron trapping sites are in fact common, not limited only to the three-phase junction due to the structural variety of mixed phase composites. For example, the photochemical studies by Linsebigler *et al.* found the trapping of electrons on anatase surfaces,^40^ which helps to enhance the photocatalytic reduction performance of anatase.^52^ Based on the DFT calculations and TEM observation, Jaroniec *et al.* have reported that the photogenerated electrons migrate to anatase(101) during the photocatalytic reduction of CO_2_.^53^ While this local trapping of electrons will further facilitate the electron–hole separation, the presence of a three-phase junction for allowing hole migration provides the fundamental mechanism for the synergistic effect in mixed oxides.

Our new model may explain why anatase needs to be the abundant phase to achieve the highest photocatalytic water splitting activity even though it is not the active site for oxygen evolution. First, the abundance of anatase phase is required to create a high concentration of A/II interface, which dominates the interfacial energy cost in forming the phase junction. Second, the hole migration is energetically more favored from anatase to A/II junction (0.3 eV) compared to that from rutile to R/II junction. This will effectively reduce the probability of charge recombination on anatase when they are created. The anatase thus should better act as a photon adsorption agent. This is consistent with the finding by Bickley *et al.* Experimentally, they showed that in P25 the majority of charge carriers will be formed in anatase, while only a small percentage of the incident radiation (<1%) is absorbed within the rutile, in spite of its large absorption coefficient.

## 5. Conclusions

This work characterizes two types of phase junction structure in between anatase and rutile for the first time, which is of great importance in photocatalysis. Type I is an unusual three-phase junction with ordered atomic structure that is the most stable phase junction; type II is the direct two-phase junction with low coordinated interfacial ions.

In the type I phase junction, a high pressure phase TiO_2_-II is present as thin layers in between rutile and anatase, and the A/II interface dominates the energy cost to form the phase junction. The lowest energy interface is identified to have the following OR, (101)_R_//(001)_II_, [101]_R_//[100]_II_ and (100)_II_//(112)_A_, [010]_II_//[110]_A_. DFT calculations with both PBE and hybrid HSE06 functionals are utilized to compute the geometrical structure and band structure of the three-phase junction. In the type II phase junction, the lowest energy interface is identified to obey the OR, (112)_A_//(101)_R_, [110]_A_//[101]_R_, where one rutile lattice is required to expand by ∼16% to fit the corresponding anatase lattice.

DFT + *U* calculations are further used to compute the thermodynamics of localized charge carriers at the two types of junctions. We find that the migration of holes is allowed while that of electrons is hindered across the three-phase junction. Thus, a new *single-way valve* model is proposed for charge carrier separation in photocatalysis, where only the holes travel across the phases while the electrons prefer to stay in the individual phases. The current model assigns the catalytic roles of anatase, rutile and phase junction in photocatalysis, *i.e.* anatase as the main photon adsorption agent and photoreduction sites, rutile as photooxidation sites and the three-phase junction for modulating the hole migration to separate electrons and holes. The results rationalize the enhanced photoactivity of anatase–rutile composite photocatalysts from the atomic level.

## Supplementary Material

Supplementary information
