# Supplementary material for: Three-phase junction for modulating electron–hole migration in anatase–rutile photocatalysts
Source: Chem Sci. 2015 Apr 7;6(6):3483–94. doi: 10.1039/c5sc00621j (PMC5659171; doi:10.1039/c5sc00621j)
Supplement: Supplementary file 1 [file SC-006-C5SC00621J-s001.pdf]

Electronic Supplementary Material (ESI) for Chemical Science.
This journal is © The Royal Society of Chemistry 2015

Three-phase Junction for Modulating Electron-Hole Migration in Anatase/Rutile Photocatalysts

Wei-Na Zhao, Sheng-Cai Zhu, Ye-Fei Li, Zhi-Pan Liu*

Collaborative Innovation Center of Chemistry for Energy Material, Shanghai Key Laboratory of Molecular Catalysis and Innovative Materials, Key Laboratory of Computational Physical Science (Ministry of Education), Department of Chemistry, Fudan University, Shanghai 200433, China

*email: zpliu@fudan.edu.cn

Structure of pure phases

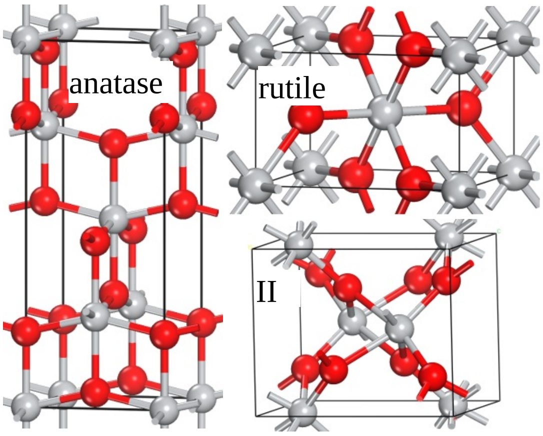

Figure S1 DFT optimized bulk structure for anatase, rutile and TiO_2-II. Ti: Grey; O: Red.

Table S1. Other models for anatase-rutile phase junction and the computed strain energy. The phase junction is constructed by joining two phases (P1 and P2) according to the OR.

	P1		P2		
OR	*a* (Å)	*b* (Å)	*a* (Å)	*b* (Å)	*S*
Direct Model					
**I**	9.68	3.81	4.65	2.97	5.98
**II**	6.57	2.97	5.54	3.81	2.36
**III**	5.54	3.81	4.65	2.97	3.07
**IV**	7.21	7.21	7.62	7.62	2.30
Indirect Model					
**V**	5.38	5.54	4.60	4.95	2.63
**VI**	7.24	6.76	5.38	5.54	3.30

* The OR include a pair of parallel crystallography planes $(hkl)_{P1}//(hkl)_{P2}$ that attach with each other (with lattice parameter ***a*** and ***b***) and a pair of parallel directions $[uvw]_{P1}//[uvw]_{P2}$. Here OR are follows. **I**: $(100)_A//(100)_R$, $[001]_A//[001]_R$; **II:** $(110)_R//(101)_A$, $[1\text{-}10]_R//[010]_A$; **III**: $(101)_A//(100)_R$, $[010]_A//[001]_R$; **IV:** $(101)_R//(001)_A$, $[11\text{-}1]_R//[100]_A$; **V:** $(112)_A//(010)_{II}$, $[1\text{-}10]_A//[001]_{II}$; **VI:** $(111)_{II}//(112)_A$; $[1\text{-}10]_{II}//[1\text{-}10]_A$.

The DFT optimized coordinates of structures

(1) anatase (car format file)

```
!BIOSYM archive 3
PBC=ON
CAR File
!DATE
PBC     3.8076      3.8076      9.6754      90.0000      90.0000      90.0000 (P1)
Ti1        0.000000000      0.000000000      0.000000000 XXXX 1          xx          Ti    0.000
Ti2        1.903778919      1.903778919      4.837690490 XXXX 1          xx          Ti    0.000
Ti3        0.000000000      1.903778919      2.418845245 XXXX 1          xx          Ti    0.000
Ti4        1.903778919      0.000000000      7.256535735 XXXX 1          xx          Ti    0.000
O1         0.000000000      0.000000000      2.005760906 XXXX 1          xx          O     0.000
O2         1.903778919      1.903778919      6.843451396 XXXX 1          xx          O     0.000
O3         0.000000000      1.903778919      4.424606151 XXXX 1          xx          O     0.000
O4         1.903778919      0.000000000      9.262296642 XXXX 1          xx          O     0.000
O5         1.903778919      0.000000000      5.250774829 XXXX 1          xx          O     0.000
O6         0.000000000      1.903778919      0.413084339 XXXX 1          xx          O     0.000
O7         1.903778919      1.903778919      2.831929584 XXXX 1          xx          O     0.000
O8         0.000000000      0.000000000      7.669620074 XXXX 1          xx          O     0.000
end
end
```

(2) rutile (car format file)

```
!BIOSYM archive 3
PBC=ON
CAR File
!DATE
```

```
PBC    4.6474    4.6474    2.9667   90.0000   90.0000   90.0000 (P1)
Ti1      0.000000000    0.000000000    0.000000000 XXXX 1      xx      Ti   0.000
Ti2      2.323719461    2.323719461    1.483358865 XXXX 1      xx      Ti   0.000
O1       1.418163368    1.418163368    0.000000000 XXXX 1      xx      O    0.000
O2       3.229275553    3.229275553    0.000000000 XXXX 1      xx      O    0.000
O3       0.905556093    3.741882829    1.483358865 XXXX 1      xx      O    0.000
O4       3.741882829    0.905556093    1.483358865 XXXX 1      xx      O    0.000
end
end
```

(3) TiO_2-II (car format file)

```
!BIOSYM archive 3
PBC=ON
CAR File
!DATE
PBC    4.5898    5.5988    4.9341   90.0000   90.0000   90.0000 (P1)
O        1.049250686    4.925464587    2.865924622 CORE 1      O      O    0.000
O        1.245658168    3.472799963    0.398898403 CORE 2      O      O    0.000
O        3.540567086    4.925464595    4.535154034 CORE 3      O      O    0.000
O        3.344159601    3.472799970    2.068127815 CORE 4      O      O    0.000
O        3.540567109    0.673378458    2.068127815 CORE 5      O      O    0.000
O        3.344159627    2.126043083    4.535154034 CORE 6      O      O    0.000
O        1.049250708    0.673378451    0.398898403 CORE 7      O      O    0.000
O        1.245658194    2.126043075    2.865924622 CORE 8      O      O    0.000
Ti      -0.000000033    4.600978034    1.233513109 CORE 9      Ti     Ti   0.000
Ti       2.294908896    3.797286524    3.700539328 CORE 10     Ti     Ti   0.000
Ti       0.000000012    0.997865012    3.700539328 CORE 11     Ti     Ti   0.000
Ti       2.294908899    1.801556522    1.233513109 CORE 12     Ti     Ti   0.000
end
end
```

(4) 3R/4II structure (car format file)

```
!BIOSYM archive 3
PBC=ON
CAR File
!DATE
PBC    4.6088    5.5723   17.4876   92.4848   90.0057   90.0170 (P1)
O        2.509544583    0.519353315    5.297063301 CORE 1      O      O    0.000
O        2.427688756    0.356233038   10.303376121 CORE 2      O      O    0.000
O        2.399604422    3.607340951   15.348495654 CORE 3      O      O    0.000
O        2.714973017    4.548712281    7.755295104 CORE 4      O      O    0.000
O        2.378766871    1.946181354   12.841945021 CORE 5      O      O    0.000
O        0.411616451    0.491349540    6.977490356 CORE 6      O      O    0.000
O        0.101776086    0.652973282   11.902743336 CORE 7      O      O    0.000
O        0.126787126    3.903639574   16.948157953 CORE 8      O      O    0.000
O        0.182892787    4.561724402    9.411720910 CORE 9      O      O    0.000
O        0.083737925    2.298671893   14.411250546 CORE 10     O      O    0.000
O        0.449079875    1.774160572    9.426680451 CORE 11     O      O    0.000
O        0.538205709   -0.485814933   14.419435367 CORE 12     O      O    0.000
O        0.231147875    3.277695901    6.964214132 CORE 13     O      O    0.000
O        0.536326084    3.438398397   11.888688446 CORE 14     O      O    0.000
O        0.507341349    1.117534532   16.961410991 CORE 15     O      O    0.000
O        2.531203986    1.760882196    7.766654292 CORE 16     O      O    0.000
O        2.847329997    4.731170803   12.827719137 CORE 17     O      O    0.000
O        2.729500758    3.303815019    5.285710344 CORE 18     O      O    0.000
O        2.813923500    3.141474821   10.287457905 CORE 19     O      O    0.000
O        2.836691401    0.818421417   15.361453867 CORE 20     O      O    0.000
O        2.480906443    0.474925822    0.356752941 CORE 21     O      O    0.000
O        2.725928719    4.639524563    2.809648554 CORE 22     O      O    0.000
O        0.414709485    0.544472191    2.018169219 CORE 23     O      O    0.000
O        0.212897414    4.627319151    4.493068269 CORE 24     O      O    0.000
O        0.423421281    1.840935883    4.504915935 CORE 25     O      O    0.000
O        0.233917466    3.328326929    2.006579167 CORE 26     O      O    0.000
O        2.524317069    1.852058986    2.826694978 CORE 27     O      O    0.000
O        2.757210036    3.259761858    0.349170029 CORE 28     O      O    0.000
Ti       1.462261823    0.099112626    8.595219260 CORE 29     Ti     Ti   0.000
Ti       1.447743524    3.434536936   13.634493225 CORE 30     Ti     Ti   0.000
Ti       3.757780258    4.950279970    6.128555174 CORE 31     Ti     Ti   0.000
Ti       3.778733723    4.586809531   11.097483653 CORE 32     Ti     Ti   0.000
Ti       3.772145466    2.287345123   16.156445930 CORE 33     Ti     Ti   0.000
Ti       1.483952689    2.160546417    6.135419977 CORE 34     Ti     Ti   0.000
Ti       1.461977190    1.807519098   11.110933132 CORE 35     Ti     Ti   0.000
```

```
Ti      1.465252847   -0.488026298   16.155726752 CORE 36   Ti   Ti   0.000
Ti      3.790702509    2.880835598    8.584757576 CORE 37   Ti   Ti   0.000
Ti      3.775002519    0.644658454   13.620204978 CORE 38   Ti   Ti   0.000
Ti      1.467778086    0.194035038    3.654191885 CORE 39   Ti   Ti   0.000
Ti      3.772298045    4.960076579    1.167437056 CORE 40   Ti   Ti   0.000
Ti      1.471761753    2.169801439    1.184107207 CORE 41   Ti   Ti   0.000
Ti      3.782714803    2.979976628    3.649400675 CORE 42   Ti   Ti   0.000
end
end
```

(5) 4A/4II structure (car format file)

```
!BIOSYM archive 3
PBC=ON
CAR File
!DATE
PBC     5.5407     5.1349     18.7819     91.0589     89.9726     90.0112 (P1)
O       0.072282787    1.208198219    5.883657967 CORE 1    O    O    0.000
O       2.717858032    2.349735345    8.247617278 CORE 2    O    O    0.000
O       1.333234497    3.196694112   10.539569351 CORE 3    O    O    0.000
O       2.759977514    2.350543475   12.797880720 CORE 4    O    O    0.000
O       5.511892937    0.595759304   15.052012177 CORE 5    O    O    0.000
O       2.852737295    2.432943224   17.360396374 CORE 6    O    O    0.000
O       1.364947664    4.945629325    8.489721112 CORE 7    O    O    0.000
O       4.117392432   -0.219238845   12.793269582 CORE 8    O    O    0.000
O       1.363357149    3.163048302   15.050695493 CORE 9    O    O    0.000
O       4.033542327   -0.133864093   17.358275031 CORE 10   O    O    0.000
O       1.272286871    3.774969667    5.876985996 CORE 11   O    O    0.000
O       4.157431757    4.914300376    8.246715054 CORE 12   O    O    0.000
O       4.105351770    3.182228046   10.744906106 CORE 13   O    O    0.000
O       1.343817316   -0.225703576   13.002469427 CORE 14   O    O    0.000
O       4.154910901    3.199257320   15.292009966 CORE 15   O    O    0.000
O       1.293927120    1.966723648    3.792695901 CORE 16   O    O    0.000
O       4.025871903    3.122719846    6.184287508 CORE 17   O    O    0.000
O       5.508699730    2.378011276    8.494147257 CORE 18   O    O    0.000
O       2.858882936    0.558747561    6.186592864 CORE 19   O    O    0.000
O       5.541268206    0.629264296   10.544868381 CORE 20   O    O    0.000
O       2.769607227    0.616744921   10.746517227 CORE 21   O    O    0.000
O       5.528439165    2.342697084   13.005523253 CORE 22   O    O    0.000
O       0.045414077    4.534983354    3.792547751 CORE 23   O    O    0.000
O       2.720672401    0.632808868   15.293340514 CORE 24   O    O    0.000
O       2.811449485    0.017366747    3.409882559 CORE 25   O    O    0.000
O       4.075224074    2.582268203    3.405396767 CORE 26   O    O    0.000
O       4.062752497    0.789345597    1.350845179 CORE 27   O    O    0.000
O       2.802986756    3.353107954    1.355594489 CORE 28   O    O    0.000
O       0.036727348    3.966357314    0.981847143 CORE 29   O    O    0.000
O       1.287674448    1.396791292    0.981090935 CORE 30   O    O    0.000
O       1.270421366    4.357262983   17.657903894 CORE 31   O    O    0.000
O       0.072117184    1.793585375   17.662138779 CORE 32   O    O    0.000
Ti      5.317627515    2.880169871    4.828979882 CORE 33   Ti   Ti   0.000
Ti      2.528508538    4.031593061    7.265932138 CORE 34   Ti   Ti   0.000
Ti      5.273951058    4.008438538    9.519072135 CORE 35   Ti   Ti   0.000
Ti      2.492251035    4.037426049   11.780943228 CORE 36   Ti   Ti   0.000
Ti      1.583350465    1.515405199   14.033837990 CORE 37   Ti   Ti   0.000
Ti      2.528004215    4.085018917   16.282965562 CORE 38   Ti   Ti   0.000
Ti      1.565224671    0.315420629    4.832699585 CORE 39   Ti   Ti   0.000
Ti      4.349639176    1.465466925    7.256627001 CORE 40   Ti   Ti   0.000
Ti      1.596179991    1.444347222    9.525357759 CORE 41   Ti   Ti   0.000
Ti      4.387881644    1.471188936   11.780215301 CORE 42   Ti   Ti   0.000
Ti      5.287616733    4.085030335   14.033096259 CORE 43   Ti   Ti   0.000
Ti      4.352112320    1.514332071   16.300619073 CORE 44   Ti   Ti   0.000
Ti      2.552677837    1.688601848    2.382814010 CORE 45   Ti   Ti   0.000
Ti      4.315797494    4.252818978    2.397128810 CORE 46   Ti   Ti   0.000
Ti      5.320190337    0.132520488   18.737317258 CORE 47   Ti   Ti   0.000
Ti      1.560669882    2.704372315   18.724845883 CORE 48   Ti   Ti   0.000
end
end
```

(6) 8A/3R structure (car format file)

```
!BIOSYM archive 3
PBC=ON
CAR File
```

```
!DATE
PBC      5.2288      5.5971      26.9288      91.8170      90.1065      90.5239 (P1)
O        0.756979989    0.020705992    19.933186489 CORE 1     O    O    0.000
O        0.718436077    3.085197728    15.087155226 CORE 2     O    O    0.000
O        3.342677083    4.366242580    17.518855271 CORE 3     O    O    0.000
O        3.342272261    1.221407786    22.392266903 CORE 4     O    O    0.000
O        0.614756974    2.265805253    24.712951390 CORE 5     O    O    0.000
O        4.793804924    2.823195072    19.946647081 CORE 6     O    O    0.000
O        2.165471090    4.033928259    22.406381774 CORE 7     O    O    0.000
O        4.762459597    0.294075719    15.116822167 CORE 8     O    O    0.000
O        2.166424430    1.579271876    17.537328738 CORE 9     O    O    0.000
O        4.980749806   -0.520439308    24.691448095 CORE 10    O    O    0.000
O        2.181064939    3.683410088    19.565663380 CORE 11    O    O    0.000
O        4.817738188   -0.688121990    21.950195909 CORE 12    O    O    0.000
O        2.391764295    0.583654740    24.551084275 CORE 13    O    O    0.000
O        4.823323310    1.560891868    26.747371847 CORE 14    O    O    0.000
O        4.777127687    2.397704726    17.133817292 CORE 15    O    O    0.000
O        3.363787076    0.871716022    19.550197630 CORE 16    O    O    0.000
O        0.734850413    2.102669390    21.957423726 CORE 17    O    O    0.000
O        3.183060350    3.372739459    24.497616065 CORE 18    O    O    0.000
O        0.779958751   -0.409860249    17.111607087 CORE 19    O    O    0.000
O        0.602857743    4.380708934    26.779287628 CORE 20    O    O    0.000
O        3.417114981    4.269878344     0.461060766 CORE 21    O    O    0.000
O        4.981105425    1.082142258     2.222913724 CORE 22    O    O    0.000
O        0.335289729    3.879492526     2.220430914 CORE 23    O    O    0.000
O        2.100057418    1.512606474     0.589269473 CORE 24    O    O    0.000
O        3.349383779    1.814464500    12.719358933 CORE 25    O    O    0.000
O        2.025932615    4.620646338    12.738179411 CORE 26    O    O    0.000
O        2.140120720    1.107676866    14.720082186 CORE 27    O    O    0.000
O        3.335361040    3.896348725    14.690549419 CORE 28    O    O    0.000
O        0.467144198    0.437794862    10.110085598 CORE 29    O    O    0.000
O        3.050622472    5.047969005     7.911808728 CORE 30    O    O    0.000
O        4.809712917    3.255044511    10.150044490 CORE 31    O    O    0.000
O        2.171691259    2.260058097     7.929550303 CORE 32    O    O    0.000
O        2.182557796    4.293464794    10.010230938 CORE 33    O    O    0.000
O        4.705092352   -0.189065250    12.256977821 CORE 34    O    O    0.000
O        3.099585276    1.486028908     9.994084328 CORE 35    O    O    0.000
O        0.708043501    2.591508336    12.218893043 CORE 36    O    O    0.000
O        3.001692132    2.584355309     3.147702657 CORE 37    O    O    0.000
O        5.019929971    4.995061617     4.638569603 CORE 38    O    O    0.000
O        0.255828244    2.222507761     4.632420641 CORE 39    O    O    0.000
O        2.302020475    5.389752717     3.164376855 CORE 40    O    O    0.000
O        4.785088750    3.408553080     7.231916643 CORE 41    O    O    0.000
O        0.483562412    0.604918212     7.208492119 CORE 42    O    O    0.000
O        2.039269154    3.698609668     5.643028497 CORE 43    O    O    0.000
O        3.261194998    0.899071753     5.623350020 CORE 44    O    O    0.000
Ti       3.484141450    2.637828823    18.575383403 CORE 45    Ti   Ti   0.000
Ti       0.875163064    3.870245989    20.996279414 CORE 46    Ti   Ti   0.000
Ti       3.570439936   -0.507808534    23.440031462 CORE 47    Ti   Ti   0.000
Ti       0.987492137    0.532473024    25.848903698 CORE 48    Ti   Ti   0.000
Ti       0.858557851    1.348081969    16.155357234 CORE 49    Ti   Ti   0.000
Ti       2.075701601   -0.179699243    18.558730956 CORE 50    Ti   Ti   0.000
Ti       4.659402195    1.052408441    20.981880905 CORE 51    Ti   Ti   0.000
Ti       1.999970679    2.267290406    23.429501051 CORE 52    Ti   Ti   0.000
Ti       4.650936123    4.134002842    16.128032296 CORE 53    Ti   Ti   0.000
Ti       4.545070744    3.297927920    25.821544359 CORE 54    Ti   Ti   0.000
Ti       4.073076409    2.945711874     1.513324031 CORE 55    Ti   Ti   0.000
Ti       1.269091132    0.205518263     1.603581291 CORE 56    Ti   Ti   0.000
Ti       3.446453266    0.057110249    13.750285973 CORE 57    Ti   Ti   0.000
Ti       2.011103133    2.853085598    13.691201449 CORE 58    Ti   Ti   0.000
Ti       3.372049953    3.376640793     8.899703889 CORE 59    Ti   Ti   0.000
Ti       0.763098192    4.362596689    11.306573999 CORE 60    Ti   Ti   0.000
Ti       1.899107375    0.570866260     8.869777414 CORE 61    Ti   Ti   0.000
Ti       4.552341608    1.562809114    11.254399126 CORE 62    Ti   Ti   0.000
Ti       3.895268485    1.097376648     3.903370140 CORE 63    Ti   Ti   0.000
Ti       1.302157206    3.889842428     3.946280045 CORE 64    Ti   Ti   0.000
Ti       4.327983902    5.196212877     6.402021410 CORE 65    Ti   Ti   0.000
Ti       0.899508990    2.417479004     6.417211969 CORE 66    Ti   Ti   0.000
end
end
```
